# Detection and Genotyping of Human Papillomavirus (HPV16/18), Epstein–Barr Virus (EBV), and Human Cytomegalovirus (HCMV) in Endometrial Endometroid and Ovarian Cancers

**DOI:** 10.3390/pathogens12030397

**Published:** 2023-03-01

**Authors:** Beniamin Oskar Grabarek, Piotr Ossowski, Justyna Czarniecka, Mateusz Ożóg, Justyna Prucnal, Ireneusz Dziuba, Aleksander Ostenda, Konrad Dziobek, Dariusz Boroń, Wojciech Peszek, Piotr Kras, Szymon Januszyk, Maciej Dąbala, Tomasz Kasela, Marcin Opławski

**Affiliations:** 1 Department of Histology, Cytophysiology and Embryology, Faculty of Medicine, Academia of Silesia, 41-055 Zabrze, Poland; 2 Department of Gynecology and Obstetrics with Gynecologic Oncology, Ludwik Rydygier Memorial Specialized Hospital, 31-826 Kraków, Poland; 3 Department of Gynecology and Obstetrics, TOMMED Specjalisci od Zdrowia, 40-662 Katowice, Poland; 4 Department of Gynecology and Obstetrics, Faculty of Medicine, Academia of Silesia, 41-055 Zabrze, Poland; 5 Gyncentrum, Laboratory of Molecular Biology and Virology, 40-851 Katowice, Poland; 6 Department of Histology and Cells Pathology, Faculty of Medicine, Medical University of Silesia in Katowice, 41-800 Zabrze, Poland; 7 Department of Microbiology, SP ZOZ Szpital Wielospecjalistyczny w Jaworznie, 43-600 Jaworzno, Poland; 8 Department of Pathology, Faculty of Medicine, Academia of Silesia, 41-055 Zabrze, Poland; 9 Faculty of Medicine, Academia of Silesia, 41-055 Zabrze, Poland; 10 Dabala Ortodoncja, 40-507 Katowice, Poland; 11 European Center of Aestheticsin Katowice, 40-055 Katowice, Poland; 12 Department of Gynecology and Obstetrics, Faculty of Medicine and Health Sciences, Andrzej Frycz Modrzewski University in Kraków, 30-705 Kraków, Poland

**Keywords:** human papillomavirus, Epstein–Barr virus, human cytomegalovirus, ovarian cancer, endometrioid endometrial cancer

## Abstract

The purpose of this study was to evaluate the relationship between human papillomavirus (HPV16/18), Epstein–Barr virus (EBV), and human cytomegalovirus (HCMV) infections and the occurrence of ovarian cancer in 48 women, of whom 36 underwent surgery and chemotherapy (group A), 12 in whom surgery was sufficient (group B), and 60 with endometroid endometrial cancer stage G1-G3 (group C), compared to patients in whom the uterus and its appendages were removed for nononcological reasons (control group). The detection of HPV, EBV, and HCMV in tumor tissue and normal tissue was performed using the real-time polymerase chain reaction (RT-PCR) technique. A statistically significantly higher risk of endometrial cancer was noted in patients infected only with HCMV (OR > 1; *p* < 0.05). In contrast, a significantly higher risk of ovarian cancer in group A was associated with HPV16, HPV18, and EBV (OR > 1; *p* < 0.05); a significantly higher risk of ovarian cancer in group B was associated with HPV18 and HMCV (OR > 1; *p* < 0.05). The obtained results suggest that HCMV infection is associated with the development of a stage of ovarian cancer when treatment can be completed with surgery alone. Meanwhile, EBV appears to be responsible for the development of ovarian cancer in more advanced stages.

## 1. Introduction

Tumors of the female genital organs are the cancers that occur most commonly in women. A significant percentage of gynecologic cancers are malignancies in which prognosis and treatment outcomes depend on the cancer stage and early diagnosis [1,2]. According to data from the National Cancer Institute, the most common malignant tumor in women in Poland is breast cancer, accounting for 22.4% of new cases. Cancers of the uterus (7%), ovary (5%), and cervix (4%) together accounted for approximately 16% of all diagnoses. This means that the aforementioned malignancies of the genital region account for approximately 40 percent of oncological cases among women [3,4,5]. Ovarian cancer causes the highest number of deaths of all gynecological cancers. The disease has an initially asymptomatic course, and cancer is most often diagnosed at a late stage (stages III–IV, according to FIGO, the International Federation of Gynecology and Obstetrics), significantly reducing the chances of a complete recovery [6,7]. Although most patients have an excellent response to first-line chemotherapy based on platinum derivatives and taxanes, recurrence usually occurs later, and drug resistance and viral susceptibility emerge [8]. The five-year survival rate for patients with stage I disease is 70–80%, but this drops to 15% for patients with stage IV ovarian cancer [9]. Late diagnosis occurs due to the asymptomatic nature of the disease in its early stages and the nonspecificity of the symptoms that appear later, which are often confused with other conditions, such as irritable bowel syndrome or premenstrual syndrome [9]. The primary mode of treatment for ovarian cancer is surgery. Primary surgery allows for the confirmation of the diagnosis, the evaluation of the stage, and the differentiation of the cancer. After the diagnosis of advanced cancer, the goal is complete cytoreduction, or removal of as much of the tumor mass as possible. The time to recurrence of ovarian cancer and the overall survival rate depend on the tumor mass that remains after surgery; complete cytoreduction results in prolonged median survival and, by reducing the tumor mass, increased response to chemotherapy. The combination of surgical treatment and chemotherapy achieves a response in more than 75% of patients. Chemotherapy can be waived after surgery if the patient’s stage IA or IB lesions are properly evaluated (i.e., if a pelvic and periaortic lymphadectomy is performed). Otherwise, patients should receive chemotherapy. Four weeks after the end of the first line of chemotherapy, the results of the treatment are evaluated. This evaluation should consist of the following: physical examination, gynecological examination, transvaginal and abdominal ultrasounds, laboratory tests, evaluation of concentrations of the markers determined during treatment, X-ray or computed tomography (CT) scan of the chest, and abdominal and pelvic CT. Since 2009, response criteria based on RECIST version 1.1 have been in effect. Patients are classified according to those who have achieved a complete response (CR), a partial response (PR), a stable disease (SD) state, and progression (PR) [10,11,12,13]. In a previous study, we conducted a clinical and molecular characterization of the same ovarian cancer patients in the context of treatment efficacy and chemotherapy resistance [14,15].

In contrast, endometrial cancer is one of the most commonly diagnosed gynecological cancers worldwide [16]. The process of tumorigenesis is associated with an imbalance of the physiologically occurring balance between the functions of proliferation and apoptosis of endometrial cells, resulting in the acquisition by tumor cells of the ability to migrate and invade neighboring tissues [17]. The factors that significantly increase the risk of developing endometrial cancer include obesity, hypertension, diabetes mellitus, postmenopausal age, and long-term exposure to estrogen that is not balanced by progesterone [18,19]. The most significant percentage of women with endometrial cancer are peri- and postmenopausal women, who often have obesity. At this time, the ovaries stop producing estrogen and progesterone and start producing androstenedione, which is converted to estrone in adipose tissue. This results in the prolonged stimulation of the endometrium by estrone, the effect of which is not balanced by progesterone, which promotes the excessive and uncontrolled proliferation of epithelial cells [20]. 

The most characteristic symptom of endometrial cancer is abnormal reproductive tract bleeding. Additional symptoms include weight loss, pain in the lower abdomen, anemia, and symptoms associated with metastasis to adjacent tissues and organs [21]. Taking the degree of the pathomorphological differentiation of endometrial cancer cells as a criterion for division, we distinguish the following categories: G1—highly differentiated (<5% solid carcinoma), G2—intermediate degree of differentiation (6–50% solid carcinoma), and G3—low degree of differentiation (>50% solid carcinoma) [22]. 

Cervical cancer belongs to a group of cancers with a specific etiology. The main causative agent of cervical cancer is chronic infection with the human papillomavirus (HPV), which has a high oncogenic potential [23,24,25]. Of the 150 types of HPV, 40 infect the female genital tract. HPV infection is limited to the basal cells of the tissue’s stratified epithelium, where viral replication occurs [26,27]. Major oncoproteins of HPV, including E6 and E7, induce cell transformation by inactivating two cellular tumor suppressor proteins, p53 and pRb, respectively [26,27]. The inactivation of pRb by E7 bypasses cellular restrictions to enter the S phase in infected cells, while the proteasomal degradation of p53 by E6 ensures cell survival by preventing apoptosis. In addition, the integration of the viral genome into the host DNA genome increases the expression of E6 and E7, leading to the hyperproliferation of tumor cells and their metastatic potential [26,27]. HPV infections are widespread in the human population and are among the most common sexually transmitted diseases [28]. HPV comprises more than 200 viral strains, among which at least 20 subtypes cause genital tract infections and have been classified as oncogenic. Especially noteworthy are types 16 and 18 [29,30]. The virulence of HPV is mainly demonstrated by the coded oncoproteins E5, E6, and E7, which cause cervical lesions of low or high malignancy (CIN-1, -2, and -3), resulting in 99.7% of squamous cells and 89% of cervical adenocarcinoma worldwide [31]. The peak of HPV infection occurs between the ages of 20 and 25. Infection is accompanied in many cases by an additional contributing factor. Pre-invasive cervical cancer is most often found in women between the ages of 30 and 40; invasive cancer is found in patients between the ages of 40 and 60. The basis of the primary prevention of cervical cancer is vaccination against the human papillomavirus. All HPV vaccines protect against HPV types 16 and 18, which account for approximately 66% of cervical cancer cases and most other cancers associated with HPV infection [25,32]. The gynecologic cancer that poses the greatest challenge to modern gynecologic oncology is ovarian cancer [33]. The most recent studies show the heterogeneous origins of ovarian cancer, with the implication that it is not a single disease entity [34]. The different origins and distinct biology of this disease make it difficult to develop effective methods for early detection [35]. There are different histological types of this cancer. The most common is serous carcinoma (70% of ovarian cancer cases), which is a low-differentiated condition in almost 90% of cases, with rapid growth and progression and a poor prognosis [36]. Viral infections contribute to many cancers worldwide and account for a significant percentage of mortality. Oncogenic viruses include the Epstein–Barr virus (EBV) and human cytomegalovirus (HCMV) [37]. HCMV infection is associated with significant morbidity and mortality in immunocompromised patients (i.e., acquired immunodeficiency syndrome), transplant patients, and cancer patients undergoing chemotherapy [38,39]. EBV was the first virus to be recognized as a carcinogen. It belongs to the Herpesviridae family and is estimated to be one of the most common human viruses [40]. The BZLF1 and LMP proteins encoded by EBV are involved in carcinogenesis. BZLF1 induces matrix metallopeptidase nine activity, resulting in the inactivation of the p53 and p56 proteins; this leads to the inhibition of tumor cell apoptosis. In addition, BRL1 causes the release of E2F and subsequently brings infected cells into the S phase of the cell cycle [41]. In addition, LMP-1 and LMP-2 are involved in activating signaling pathways associated with the hyperproliferation of infected cells and the induction of resistance of these cells to treatment [41,42].

According to previous studies, viral infections, including those caused by HPV, EBV, HBV, and hepatitis C virus (HCV), are responsible for 15–20% of gynecologic cancers [43,44].

The purpose of our prospective study was to evaluate the relationship between viral infections with HCMV, EBV, and HPV 16 and 18 and the occurrence of ovarian cancer and endometrial cancer in patients at different stages of differentiation, relative to patients who had their uterus and its appendages removed for nononcological reasons (the control group); additionally, we estimated the risk of a given type of cancer depending on infection with HPV16/18, HCMV, and EBV.

## 2. Materials and Methods

### 2.1. Ethics 

The present study was performed following the 2013 Declaration of Helsinki guidelines on human experimentation. It is impossible to identify patients individually in this study or in the database. Informed consent was obtained from all patients. Approval from the Bioethical Committee operating at the Regional Medical Chamber in Kraków (approval no. 185/KBL/OIL/2020 and 186/KBL/OIL/2020, dated 20 September 2020) was obtained for this study.

All of the described procedures were performed in the Gynecology and Obstetrics Department with the Gynecology Oncology and Clinical Oncology Unit of Ludwik Rydygier Specialist Hospital in Kraków, Poland.

### 2.2. Subjects

#### 2.2.1. Patients Diagnosed with Endometroid Carcinoma of the Endometrium

The study included patients diagnosed with stage I–IV ovarian cancer (n = 48), for whom the median age was 63 years, and endometroid endometrial cancer of the histopathological stage G1–G3 (n = 60), for whom the median age was 65 years. Among women with endometroid endometrial cancer, based on histopathological examination, 3 subgroups were distinguished according to the degree of differentiation: 15 samples in G1, 15 samples in G2, and 15 samples in G3. In all cases, surgery was performed, which included the radical removal of the uterus and the removal of the pelvic and preaortic lymph nodes. This type of action is the gold standard in the treatment of this cancer.

#### 2.2.2. Patients Diagnosed with Ovarian Cancer

In the group of patients with ovarian cancer type II, 36 women were distinguished (group A) in whom surgical treatment was supplemented with first-line chemotherapy, according to current standards (n = 5 patients with stage I cancer; n = 5 patients with stage II cancer; n = 25 patients with stage III cancer; n = 1 patient with stage IV cancer).

In contrast, in the 12 remaining patients diagnosed with type I ovarian cancer (group B), 11 patients were in stage I, and 1 woman was diagnosed with stage IV. In this group, treatment ended with surgery. According to the recommendations of the Polish Society of Gynecologic Oncology for the diagnosis and treatment of ovarian cancer, postoperative chemotherapy was abandoned due to the low grade of the tumor lesions, as determined after a full surgical evaluation of the lesions’ progression (i.e., pelvic and periaortic lymphadenectomy performed) [12].

Surgical treatment included the removal of the uterus with adnexa, the appendix, the mesh (nonmesh), pelvic minor lymph nodes, and pelvic and pre-aortic minor lymph nodes. On the other hand, chemotherapy was administered with cisplatin, according to current standards [10,11,12,13].

#### 2.2.3. Control Group

The control was 50 women (n = 50), with a median age of 49 years, in whom the uterus and adnexa had been surgically removed for nononcological reasons.

The characteristics of the participants in both the study group and the control group are presented in Table 1.

### 2.3. Tissue Collection

Tumor-lesioned and normal tissues were collected during surgery and preserved in 4% buffered formalin (POL-AURA, catalog number Morag, Poland; 114321729#10L) for histopathological analysis and, later, RNA, for molecular evaluation. The samples for molecular testing were stored at −80 °C (Thermo Fisher Scientific, Waltham, MA, USA; catalog number AM7020) until the assays were performed.

### 2.4. Deoxyribonucleic Acid (DNA) Extraction

The DNA extraction was performed using the commercially available QIAamp DNA Mini Kit and Blood Mini Kit (Qiagen GmbH, Hilden, Germany) according to the manufacturer’s recommendations. The DNA extracts were evaluated qualitatively by performing electrophoretic separation in a 1% agarose gel and quantitatively (Nanodrop®, Thermo Fisher Scientific, Waltham, MA, USA) by assessing the concentration of the extract at 260 nm and its purity according to the ratio of absorbance from 260 to 280 nm (standard 1.8–2.0).

#### 2.4.1. HPV Detection in Tissues

The detection of HPV in tissues was performed using a real-time polymerase chain reaction (RT-PCR) based on a single-plate test for genotypes 16 and 18, as recommended by Lindh et al. [45] and Igerslev et al. [46]. RT-PCR was performed using a Roche®LC480 Lightcycler. The volume of the reaction mixture was 50 μL, and the thermal profile of the reaction was as follows: 95 °C for 5 min, 35 cycles of 95 °C for 30 s, 60 °C for 30 s, 72 °C for 60 s, and a final extension step of 5 min. Glyceraldehyde-3-phosphate dehydrogenase (GAPDH) was used as an endogenous control in the reaction. To validate any positive results, a negative control without a template and nucleic acid were included in each run. As the original study design recommended, a cycle threshold of <35 was taken as a negative result.

#### 2.4.2. HCMV Detection in Tissues

A commercially derogated artus CMV TM PCR kit (Qiagen GmbH, Hilden, Germany) was used according to the manufacturer’s recommendations for HCMV detection in tumor and normal tissues, which allows for the specific amplification of the 105 bp region of the HCMV genome. RT-PCR was performed on a Roche®LC480 Lightcycler. The volume of the reaction mixture was 50 μL, and the thermal profile of the reaction was as follows: 95 °C for 5 min, 35 cycles of 95 °C for 30 s, 60 °C for 30 s, 72 °C for 60 s, and a final extension step of 5 min. Glyceraldehyde-3-phosphate dehydrogenase (GAPDH) was used as an endogenous control in the reaction. To validate any positive results, a negative control without a template and nucleic acid were included in each run. A result of >5 copies/μg DNA was taken as a positive result. 

#### 2.4.3. EBV Detection in Tissues

The RealStar® EBV PCR Kit 1.0 y RealStar® EBV PCR Kit 1.2 (Barcelona, Spain), approved by CE-IVD, was used for EBV detection in the test and control samples. RT-PCR was performed on a Roche®LC480 Lightcycler. The volume of the reaction mixture was 50 μL, and the thermal profile of the reaction was as follows: 95 °C for 5 min, 35 cycles of 95 °C for 30 s, 60 °C for 30 s, and 72 °C for 60 s, and a final extension step of 5 min. Glyceraldehyde-3-phosphate dehydrogenase (GAPDH) was used as an endogenous control in the reaction. In order to validate any positive results, a negative control without a template and nucleic acid were included in each run. According to the manufacturer’s recommendations, the analytical sensitivity was 1.1 copies/μL; 95% confidence interval (CI): 0.578–3.253 copies/μL.

### 2.5. Statistical Analysis

The statistical calculations were made using the Social Science Statistics webpage [47].

The *p*-value was calculated using a chi-square test with a Yates correction (χ^2^), and a *p*-value below 0.05 was considered significant (*p* < 0.05). In turn, the measurable data are presented as the mean ± standard deviation (SD). Logistic regression was used to calculate the odds ratios (ORs) and their corresponding 95% confidence interval (95% CI) by comparing the case group to the control group. If OR ≈ 1, the likelihood of the event in both groups was similar. If OR < 1, there was a lower probability of the event occurring in the study group (i.e., the reference group). If OR > 1, there was a greater chance of a given event occurring in the study group (i.e., the reference group).

## 3. Results

### 3.1. Relationship between HPV, CMV, and EBV Infection and Endometroid Endometrial Cancer

The statistical analysis showed no statistically significant relationship between HPV16/18, HMCV, or EBV infection and the occurrence of endometrial cancer in the evaluated patient population (*p* > 0.05). In each of the stages of endometrial cancer, the number of HPV16-positive cases was the same (n = 2; 13.33%); meanwhile, for HPV-18, the highest proportion of positive results was recorded for patients with endometrial cancer in stage G2 (n = 4; 26.67%). We also found the highest number of HCMV-positive results in G2 patients (n = 6; 40%). EBV, on the other hand, was most common in patients with stage G1 endometrial cancer (n = 2; 13.33%). The detailed results of the chi-square analysis are shown in Table 2.

### 3.2. Relationship between HPV, CMV, and EBV Infection and Ovarian Cancer

The statistical analysis showed only one statistically significant relationship between HPV16/18, HMCV, or EBV infection and stage III ovarian cancer; this was found in group A (*p* = 0.020). For the other stages of ovarian cancer, no such relationship was confirmed (*p* > 0.05). The highest percentage of HPV16-positive (n = 3; 60%), EBV-positive (n = 2; 40%), and HCMV-positive (n = 2; 40%) results was recorded in group A at stage II. In contrast, the highest proportion of HPV18-positive cases was recorded in group A for stage III (n = 10, 40%) and in group B for stage I (n = 5; 45.46%). However, the small number of patients in each subgroup should be considered when interpreting the results. No chi-square analysis was performed for stage IV patients in groups A and B due to the insufficient group sizes. The detailed results of the chi-square analysis are shown in Table 3.

### 3.3. Risk Assessment of Endometroid Endometrial Cancer and Ovarian Cancer in Relation to HPV16/18, HCMV, and EBV Infection

A statistically significantly higher risk of endometrial cancer was noted in patients infected with HCMV alone (OR > 1; *p* < 0.05). When infected with one or more of the other assessed viruses, the risk of endometrial cancer was higher (OR > 1) but not significant (*p* > 0.05). Our statistical analysis showed that infection with HPV16, HPV18, HCMV, or EBV increases the risk of endometrial and ovarian cancer, but it transpired that not all of these results were significant (*p* > 0.05). A statistically significantly higher risk of endometrial cancer was observed in patients infected with HCMV alone (OR > 1; *p* < 0.05). In contrast, a significantly higher risk of ovarian cancer qualifying for surgery and chemotherapy (group A) was associated with infection with one of the assessed oncogenic viruses: HPV16 (OR > 1; *p* < 0.05), HPV18 (OR > 1; *p* < 0.05), and EBV (OR > 1; *p* < 0.05). Meanwhile, a significantly higher risk of ovarian cancer in the good prognosis group (i.e., when chemotherapy can be waived (group B)), was associated with HPV18 (OR > 1; *p* < 0.05) and HCMV infection (OR > 1; *p* < 0.05). Table 4 details the risk scores for developing endometrial or ovarian cancer according to oncogenic virus infection.

### 3.4. Coinfections of HPV16/18, CMV, and EBV in Patients with Endometroid Endometrial Cancer and Ovarian Cancer, as Well as the Control Group

We noted no co-infection with HPV16/18, CMV, and EBV in the control group (n = 0; 0%). In contrast, we recorded two co-infections of HPV18 and CMV among patients with endometrial cancer, specifically in patients with stage G3 endometrial cancer (n = 2; 13.33%). Moreover, we noted one co-infection with HCMV and EBV among ovarian cancer patients in subgroup B stage I (n = 1; 6.67%).

## 4. Discussion

In this work, the patients diagnosed with ovarian cancer based on molecular criteria were assigned to group A (type II ovarian cancer) or group B (type I ovarian cancer) [48]. Type I develops from benign lesions or cancers of limited malignancy and accounts for approximately one-third of all cases. It develops as a tumor in the ovary and, after some time, spreads to the peritoneal cavity. Histologically, it is usually endometrial or mucinous. Type I has a better prognosis than type II, which develops as a malignant tumor from the beginning. Type II ovarian cancer is often detected at stage III or IV. Relatively often, no tumor is observed within the ovary itself, and the disease spreads to the peritoneal cavity. Histologically, it is usually a poorly differentiated serum carcinoma with a poor prognosis [49].

In our study, all patients diagnosed with endometrial cancer were postmenopausal and overweight or obese. In our study, we showed a higher prevalence of HPV18 compared to HPV16 in patients with endometrial cancer (10 cases vs. 6 cases, respectively), which is consistent with the observations of Bruni et al., who showed that HPV16 is more common in patients younger than 60. HPV18, on the other hand, is more prevalent among women over the age of 60 [50]. In our study, all patients were at least 60 years old. Also Abu-Labad et al. and tan et al. showed a higher prevalence of HPV18 than HPV16 among women with endometrial cancer, indicating that the prevalence of infection caused by HPV16 and HPV18 genotypes is also population dependent [51,52]. 

Our statistical analysis showed that infection with HPV16, HPV18, HCMV, or EBV increases the risk of endometrial and ovarian cancer, but not all of our results were significant (*p* < 0.05). A statistically significantly higher risk of endometrial cancer was observed in patients infected with HCMV alone (OR > 1; *p* < 0.05). In contrast, a significantly higher risk of ovarian cancer qualifying for surgery and chemotherapy (group A) was associated with infection with one of the assessed oncogenic viruses: HPV16 (OR > 1; *p* < 0.05), HPV18 (OR > 1; *p* < 0.05), and EBV (OR > 1; *p* < 0.05). Moreover, a significantly higher risk of ovarian cancer in the group with a good prognosis (i.e., when chemotherapy can be waived (group B)) was associated with HPV18 (OR > 1; *p* < 0.05) and HCMV infections (OR > 1; *p* < 0.05). The demonstration, in the statistical analysis performed, of a statistically significantly higher risk of ovarian cancer type I (group B) when infected only with the HPV16 genotype and for ovarian cancer type II (group A) with both the HPV16 and HPV18 genotypes may be due, as we pointed out in the case of endometrial cancer, to the age of the study population and the geographic distribution of the different HPV genotypes [50,51,52]. 

In addition, the result of the statistical analysis is affected by the size of the sample for which the calculations were performed. Thus, if the study population had been larger, it is not impossible that we would have shown a statistically significant risk between HPV16 infection and the development of type I ovarian cancer for women with type I ovarian cancer. On the other hand, Roos et al. showed that only HPV18 infection significantly increases the risk of ovarian cancer, precisely type I as opposed to HPV 16 [53]. A higher prevalence of HPV16 than HPV18 has been reported, for example, in cervical cancer and head-and-neck neoplasms [54] and the opposite trend in glandular carcinomas [55,56]. 

The HPV16 and 18 genotypes are among the subtypes with high oncogenic potential (HR-HPV) and account for approximately 96% of cervical cancer cases. The frequency of HPV detection in women with ovarian cancer varies geographically [28,57]. HPV infection is recognized as a significant cause of cervical cancer. Studies have shown that chronic and recurrent HPV infections are closely associated with gynecologic malignancies, such as endometrial and cervical cancers [58]. 

A similar study was conducted in China at Harbin Medical University Cancer Hospital from 2006–2016 [59]. Yang et al. studied the prevalence of HPV in 310 ovarian cancer patients using RT-PCR and immunohistochemical staining to detect the p16 protein in tumor biopsy specimens. The overexpression of the p16 protein and HPV DNA was present in 100 (32.3%) of the 310 cases and correlated with high levels of PD-L1 expression. These researchers found that the overexpression of p16 was significantly more common in patients with active HPV infections [59]. 

Additionally, Hammou et al. evaluated the prevalence of HPV16/18 DNA in a group of 70 patients with confirmed ovarian cancer. Of the 70 ovarian cancer samples analyzed, HPV DNA was detected in 11.42% of cases (8/70). Only two HPV genotypes were identified, namely, HPV16 (87.5%) and HPV31 (12.5%). A recent study on a population of Moroccan women showed a strong correlation between the presence of HPV16/18 DNA and the development of ovarian cancer [60]. However, Ingersley et al. did not show an association between HPV infection and the occurrence of ovarian cancer in a Caucasian population (*p* > 0.05); our study also focused on Caucasian women [46]. The discrepancy between the findings of Ingersley et al. and those reached in the present study may have been caused by the different methods used to prepare the tumor tissues for molecular analysis and by the more significant number of samples used in the former study [46].

Nevertheless, it should be noted that the available literature does not show a relationship between HPV16/18 infection and endometrial cancer [61,62]; this was also confirmed by our analysis (*p* > 0.05). Bouziyane et al. evaluated HPV DNA in 93 endometrial cancer samples. These authors detected the HPV16, HPV30, HPV6, and HPV11 genotypes and viral DNA in only 11 of 71 samples (15.49%), indicating no association between HPV infection and endometrial cancer [61]. 

Using an in situ hybridization technique and PCR methods, Karadayi et al. did not note the presence of HPV in any of the 60 endometrial cancer samples under consideration [62].

Recent studies indicate that HCMV, which is estimated to have infected nearly 50% of the world’s population, may play a role in inducing carcinogenesis through its immunomodulatory effects. In healthy individuals, primary HCMV infection is essentially asymptomatic. The virus then establishes a lifelong chronic latency, mainly in hematopoietic progenitor cells in the bone marrow, with periodic reactivation from latency, which is often characterized by the shedding of pro-inflammatory cytokines (i.e., a cytokine storm) [63]. Additionally, it is suggested that HCMV-induced infection is associated with the induction and progression of ovarian cancer. The results of our study confirm that HCMV infection increases the risk of type I ovarian cancer (group A) to a statistically significant degree. At the same time, we did not establish the relationship between HMCV infection and the type II ovarian cancer risk (group B). Therefore, a history of HMCV disorder may be associated with more advanced forms of ovarian cancer and a worse prognosis. Nevertheless, the fact that only a small number of samples represented the remaining stages of ovarian cancer should be taken into account, as this factor probably influences the results we obtained [63]. 

The results of our study support the findings reported by Yin et al. [64]. These researchers determined the expression of HCMV IE protein and HCMV tegument protein pp65 in 66 formalin-fixed paraffin-embedded ovarian cancer specimens, compared to 30 benign ovarian cystadenoma specimens, using immunohistochemical staining. The expression of both proteins was significantly more frequent in ovarian cancer specimens than in the controls [64]. Thus, HCMV infection is a potential risk factor for the development of ovarian cancer and is associated with an unfavorable prognosis. The association between HCMV and clinical outcomes underscores the need for further research into the oncomodulatory role of HCMV in ovarian cancer [64]. 

Our study shows that EBV infection increases the risk of developing endometrial and ovarian cancers, with a statistically significantly higher risk for ovarian cancer alone (OR > 1; *p* < 0.05) [65]. Our results are consistent with the observations of Shokouh et al., who evaluated the association between EBV and HPV infections and ovarian cancer [66]. They found a significantly higher frequency of HPV and EBV infection in patients with malignant ovarian cancer compared to a control group and to a group of women with benign ovarian cancer (*p* < 0.05). Additionally, Littmann et al. showed that the risk of developing ovarian cancer is higher the later the first EBV infection occurs [67]. In addition, Pandya et al. showed significantly higher levels of expression of EBV miR-BART7 in tumor tissue compared to normal tissue, confirming the oncogenic role of EBV (*p* > 0.05) [68].

The strengths of our study include the detailed description of the characteristics of the study and control groups, taking into account the histopathological stages of endometrial cancer and ovarian cancer. Moreover, we detected viral DNA in the obtained biopsy specimens secured in RNA later, which prevents the degradation of nucleic acids. This is important because the detection of viral genetic material placed in formalin-fixed and paraffin-embedded biopsy specimens is less optimal and does not fully protect the nucleic acid from degradation [69,70]. The main strength of our study lies in the detection of the genetic material of three viruses (EBV, HCMV, and HPV16/18) with a confirmed role in the carcinogenesis of gynecological cancers.

The first limitation of our study is its single-center nature and the relatively small number of samples used, especially in the case of ovarian cancer. Therefore, the results obtained should be interpreted with caution. Second, it is essential to remember that long periods may elapse between the onset of EBV, HCMV, and HPV16/18 infection and the development of a primary neoplastic lesion. Schiffmann et al. showed that the average time between active disease caused by HPV and the onset of cervical cancer is 15–20 years [71]. 

Meanwhile, Hoots et al. indicated that HPV DNA detection is only possible in malignant lesions of cervical and rectal cancer and not in benign lesions [72]. Third, this study would benefit from confirming virus infection by determining IgG and IgM class antibody titers in serum samples from women with endometrial cancer and ovarian cancer and comparing these results to control samples.

## 5. Conclusions

Our statistical analysis showed that infection with HPV16, HPV18, HCMV, and EBV increases the risk of endometrial and ovarian cancers, but not all of our results were significant (*p* < 0.05). However, a statistically significantly higher risk of endometrial cancer was noted in patients infected with HCMV (OR = 19.91; *p* < 0.05). When infected with one or more of the other viruses under consideration, the risk of endometrial cancer was higher (OR > 1) but not significantly so (*p* > 0.05). In contrast, a significantly higher risk of ovarian cancer qualifying for surgery and chemotherapy (group A) was associated with infection with one of the assessed oncogenic viruses: HPV16 (OR > 1; *p* < 0.05), HPV18 (OR > 1; *p* < 0.05), or EBV (OR > 1; *p* < 0.05). Moreover, a significantly higher risk of ovarian cancer in the good prognosis group (i.e., when chemotherapy can be waived (group B)) was associated with HPV18 (OR > 1; *p* < 0.05) and HCMV infections (OR > 1; *p* < 0.05). Thus, the results suggest that HCMV infection is associated with the development of stages IA and IB ovarian cancer when treatment can be terminated with surgery. Meanwhile, EBV appears to be responsible for the development of ovarian cancer in more advanced stages. Taking into account the limitations of this study, further studies involving larger groups are necessary. 

## Figures and Tables

**Table 1:** Clinical and demographic data of the control group and of patients with endometrial and ovarian cancers.

Group		Age (years)	Body Weight (kg)	Height (cm)	BMI (kg/m^2^)	Menopause	Age at Menopause (years)
Control group		47 ± 3	68.9 ± 5.67	158 ± 6	26.87 ± 5.61 Overweight	11 (22%)	45 ± 7
Endometroid endometrial cancer	G1	71 ± 5	67.89 ± 5.12	154 ± 5	29.98 ± 4.56 Overweight	15 (100%)	51 ± 6
	G2	67 ± 6	66.98 ± 4.44	156 ± 4	30.12 ± 4.31 obesity	15 (100%)	49 ± 4
	G3	72 ± 5	67.18 ± 2.34	153 ± 6	28.54 ± 4.31 Overweight	15 (100%)	50 ± 6
Ovarian cancer group A	I	65 ± 4	64.5 ± 6.1	155 ± 7	23.98 ± 3.41 Normal weight	3 (60%)	51 ± 3
	II	62 ± 5	63.45 ± 3.41	152 ± 4	24.56 ± 4.32 Normal weight	1 (20%)	52 ± 2
	III	65 ± 7	64.98 ± 4.87	158 ± 8	24.41 ± 3.45 Normal weight	19 (76%)	51 ± 3
	IV	68	68	154	28.7 Overweight	Yes	57
Ovarian cancer group B	I	51 ± 6	65.13 ± 3.21	159 ± 2	25.67 ± 5.66 Overweight	7	52 ± 3
	IV	48	56	156	23 Normal weight	No	-

Data are presented as the mean ± standard deviation (SD). BMI, body mass index; G, grading.

**Table 2:** Number of HPV16/18-, HMCV-, and EBV-positive samples in the group of patients with endometroid endometrial cancer and in the control group.

Group		HPV16	HPV18	HCMV	EBV	χ^2^ (*p*-Value)
Control group (n = 50)		3 (6%)	4 (8%)	1 (2%)	1 (2%)	3.141 (0.370)
Endometroid endometrial cancer (n = 45)	G1 (n = 15)	2 (13.33%)	3 (23.08%)	2 (13.33%)	2 (13.33%)	0.392 (0.942)
	G2 (n = 15)	2 (13.33%)	4 (26.67%)	6 (40%)	1 (6.67%)	5.7938 (0.122)
	G3 (n = 15)	2 (13.33%)	3 (23.08%)	5 (33.33%)	1 (6.67%)	3.896 (0.273)

HPV16/18, human papillomavirus genotyping 16/18; HMCV, human cytomegalovirus; EBV, Epstein–Barr virus; χ^2^, chi-squared test results; *p*, statistically significance level; n, number of cases (%); G, grading.

**Table 3:** Number of HPV16/18-, HMCV-, and EBV-positive samples in ovarian cancer patients and in the control group.

Group		HPV16	HPV18	HCMV	EBV	χ^2^ (*p*-Value)
Control group (n = 50)		3 (6%)	4 (8%)	1 (2%)	1 (2%)	3.141 (0.370)
Ovarian cancer (group A; n= 36)	I (n = 5)	3 (60%)	1 (20%)	1 (20%)	1 (20%)	0.800 (0. 849)
	II (n = 5)	3 (60%)	1 (20%)	2 (40%)	2 (40%)	1.667 (0.644)
	III (n = 25)	11 (44%)	10 (40%)	2 (8%)	6 (24%)	9.859 (0.020)
	IV (n = 1)	0	0	0	0	-
Ovarian cancer (group B; n = 12)	I (n = 11)	3 (27.28%)	5 (45.46%)	4 (36.67%)	1 (9.09%)	3.440 (0.329)
	IV (n = 1)	0	0	0	0	-

HPV16/18, human papillomavirus genotyping 16/18; HMCV, human cytomegalovirus; EBV, Epstein–Barr virus; χ^2^, chi-squared test results; *p*, statistically significance level; n, number of cases (%).

**Table 4:** Estimated significant (*p* < 0.05, one-sided) odds ratio of the risk of cancer incidence vs. virus infection (multivariate regression).

Comparison	Virus	OR	95% Cl	*p*-Value
Endometroid endometrial cancer vs. control group	HPV16	2.410	0.566–10.270	0.234
	HPV18	3.290	0.950–11.355	0.060
	HCMV	19.910	2.481–159.690	0.005
	EBV	4.780	0.514–44.469	0.169
Ovarian cancer (group A) vs. control group	HPV16	37.600	4.664–303.078	0.0007
	HPV18	5.750	1.673–19.762	0.0055
	HCMV	7.903	0.881–70.8778	0.0647
	EBV	16.333	1.963–135.906	0.0098
Ovarian cancer (group B) vs. control group	HPV16	3.620	0.651–20.072	0.1470
	HPV18	5.227	1.202–22.740	0.0275
	HCMV	16.333	1.670–159.760	0.0164
	EBV	3.267	0.1930–55.441	0.4126

HPV16/18, human papillomavirus genotyping 16/18; HMCV, human cytomegalovirus; EBV, Epstein–Barr virus; OR, odds ratio; 95% CL, 95% confidence interval.

## Data Availability

The data used to support the findings of this study are included in the article. The data will not be shared due to the fact of third-party rights and commercial confidentiality.
